# Supplementary material for: Kastor and Polluks polypeptides encoded by a single gene locus cooperatively regulate VDAC and spermatogenesis
Source: Nat Commun. 2022 Feb 28;13:1071. doi: 10.1038/s41467-022-28677-y (PMC8885739; doi:10.1038/s41467-022-28677-y)

SUPPLEMENTARY INFORMATION

Kastor and Polluks polypeptides encoded by a single gene locus cooperatively regulate VDAC and spermatogenesis

Shintaro Mise, Akinobu Matsumoto, Keisuke Shimada, Toshiaki Hosaka, Masatomo Takahashi, Kazuya Ichihara, Hideyuki Shimizu, Chisa Shiraishi, Daisuke Saito, Mikita Suyama, Tomoharu Yasuda, Toru Ide, Yoshihiro Izumi, Takeshi Bamba, Tomomi Kimura-Someya, Mikako Shirouzu, Haruhiko Miyata, Masahito Ikawa & Keiichi I. Nakayama

SUPPLEMENTARY FIGURES

UNCROPPED IMAGES OF WESTERN BLOT ANALYSIS

SUPPLEMENTARY FIGURES

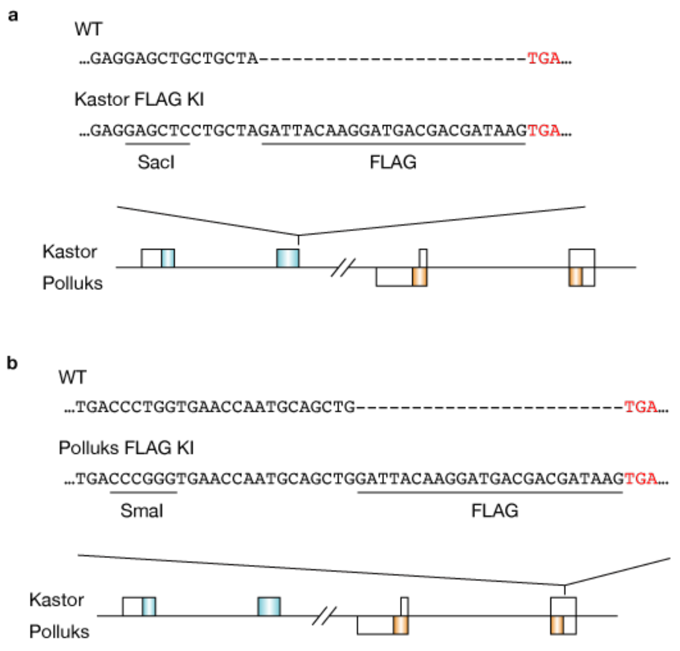

Supplementary Fig. 1 Generation of FLAG-tag knock-in mice. Schematic representations of the WT and mutant alleles for knock-in (KI) mice that harbor a FLAG epitope tag sequence at the COOH-terminus of Kastor (**a**) or Polluks (**b**) are shown. Exons are denoted by boxes. ORFs of Kastor and Polluks are represented by blue and orange boxes, respectively. Red characters indicate stop codons.

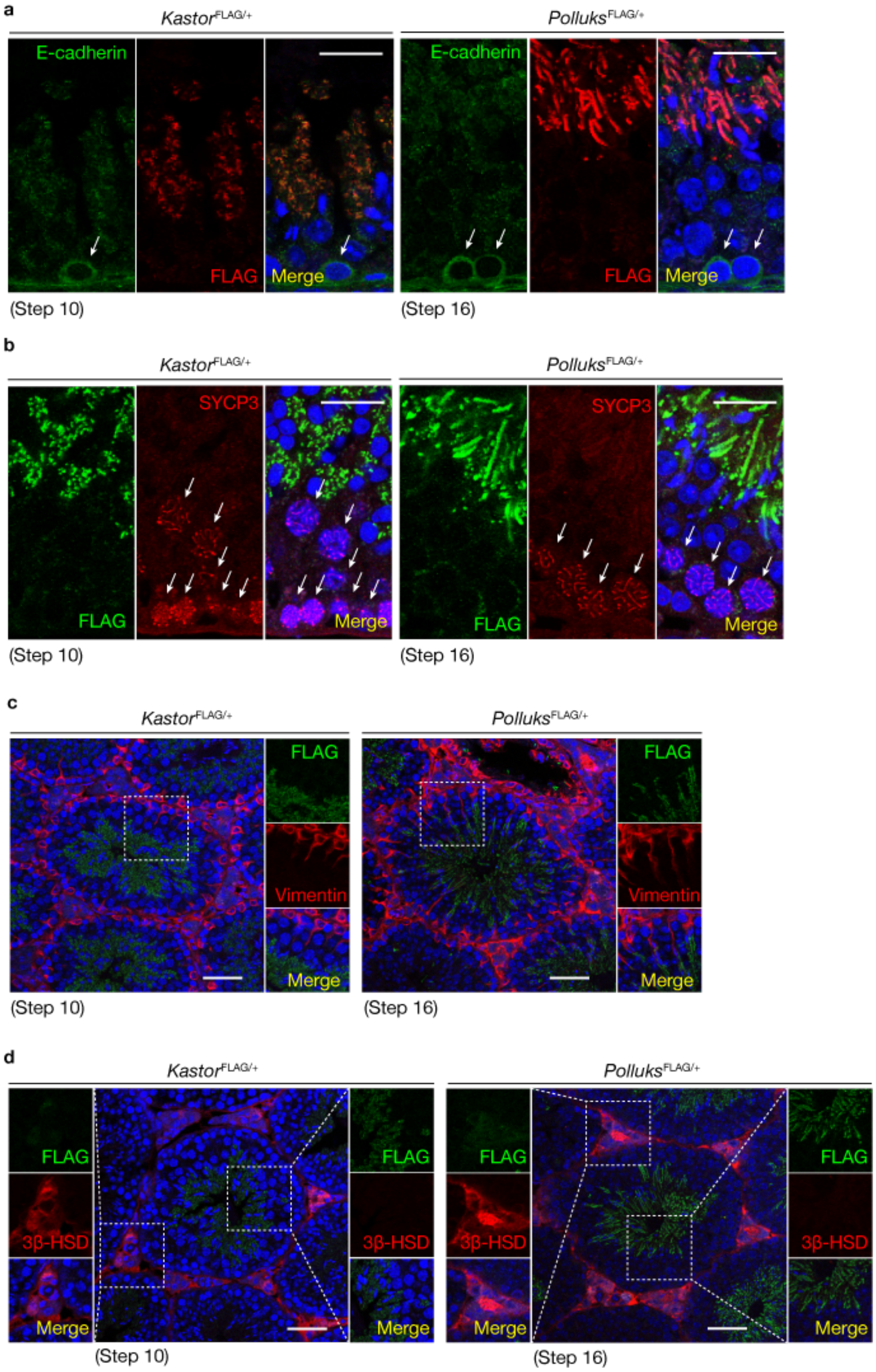

Supplementary Fig. 2 Expression patterns of Kastor and Polluks in the testis. The testis of adult *Kastor*$^{FLAG/+}$ or *Polluks*$^{FLAG/+}$ mice was subjected to immunofluorescence staining with antibodies to FLAG and those to E-cadherin (**a**), to SYCP3 (**b**), to vimentin (**c**), or to 3β-HSD (**d**). Nuclei were stained with DAPI (blue). Arrows in (**a**) and (**b**) indicate E-cadherin–positive cells and SYCP3–positive cells, respectively. The boxed areas of the large images in (**c**) and (**d**) are shown at higher magnification in the

corresponding smaller images. The steps of spermiogenesis represented in all images are indicated. Scale bars, 20 µm (**a**, **b**) or 50 µm (**c**, **d**).

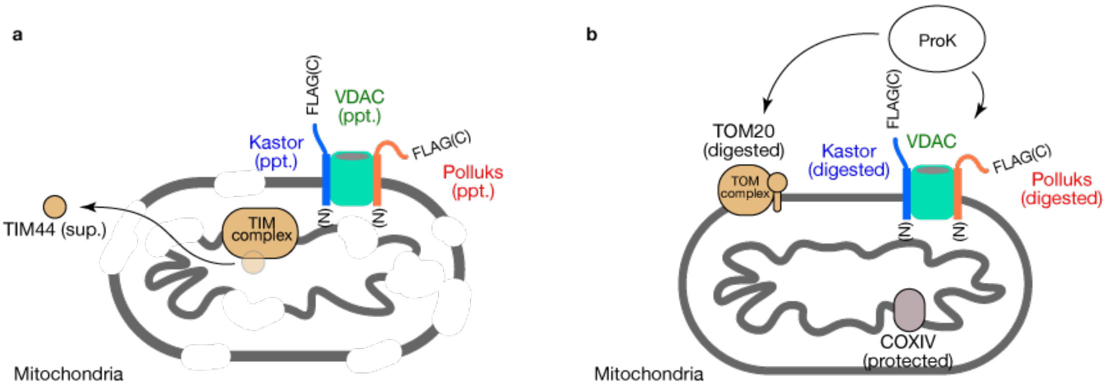

Supplementary Fig. 3 Topological analysis of Kastor and Polluks. Schematic representations of the topology assays corresponding to Figure 3e and 3f (**a**) or to Figure 3g and 3h (**b**) are shown.

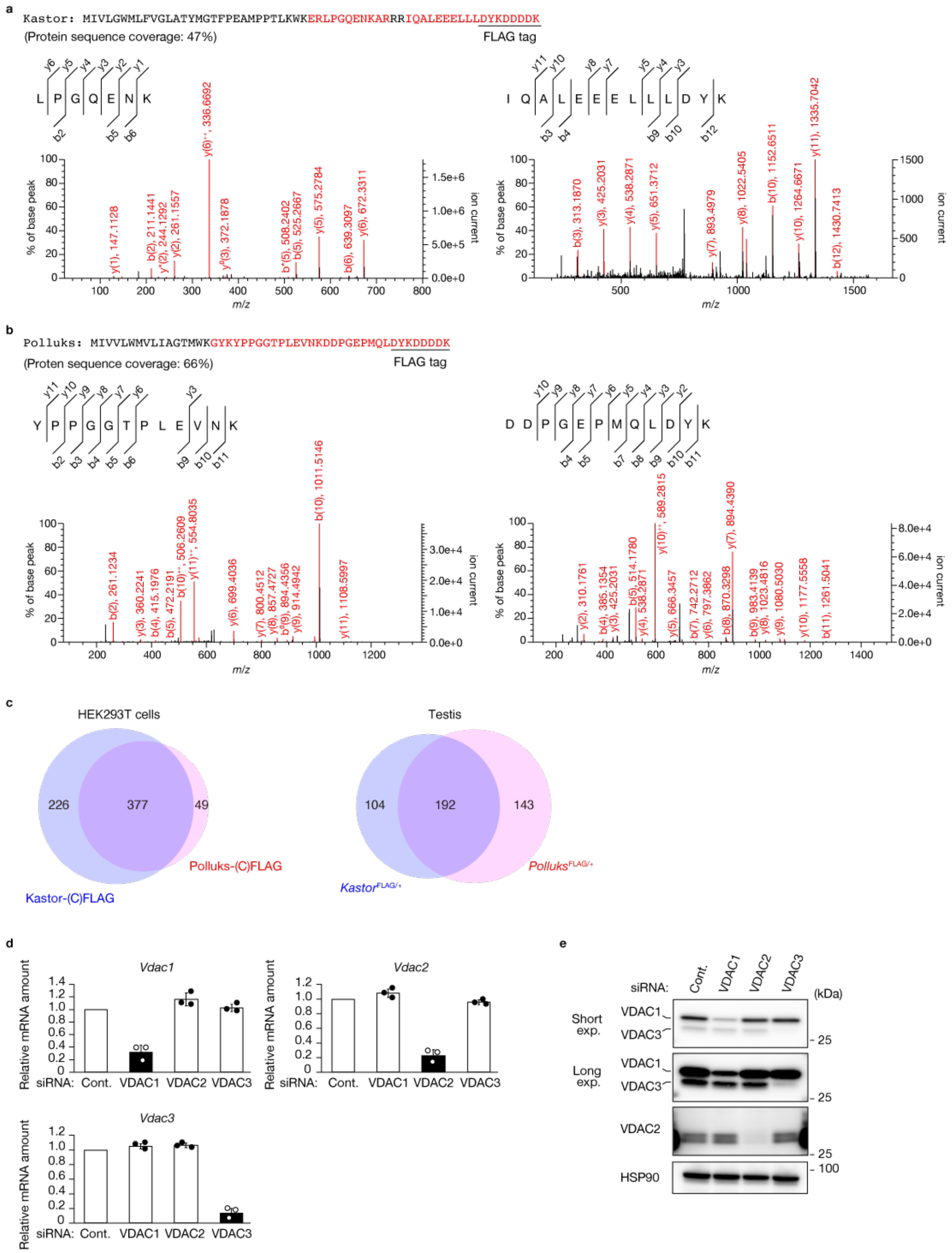

Supplementary Fig. 4 Binding proteins for Kastor and Polluks as well as validation of VDAC antibody specificity. a, **b** MS/MS spectra and protein sequence coverage for Kastor (**a**) and Polluks (**b**). Identified peptide sequences are shown in red characters. **c**

Venn diagrams showing the numbers of binding proteins for Kastor or Polluks identified in HEK293T cells transiently expressing Kastor-(C)FLAG or Polluks-(C)FLAG or in the testis of *Kastor*$^{FLAG/+}$ and *Polluks*$^{FLAG/+}$ mice. **d** RT and quantitative PCR (qPCR) analysis of *Vdac1*, *Vdac2*, and *Vdac3* mRNA abundance in Neuro2A cells transfected with control (Cont.), VDAC1, VDAC2, or VDAC3 siRNAs. Data are means ± s.d. (n = 3 biological replicates). **e**, Immunoblot analysis of Neuro2A cells transfected with the indicated siRNAs. Source data are provided as a Source Data file.

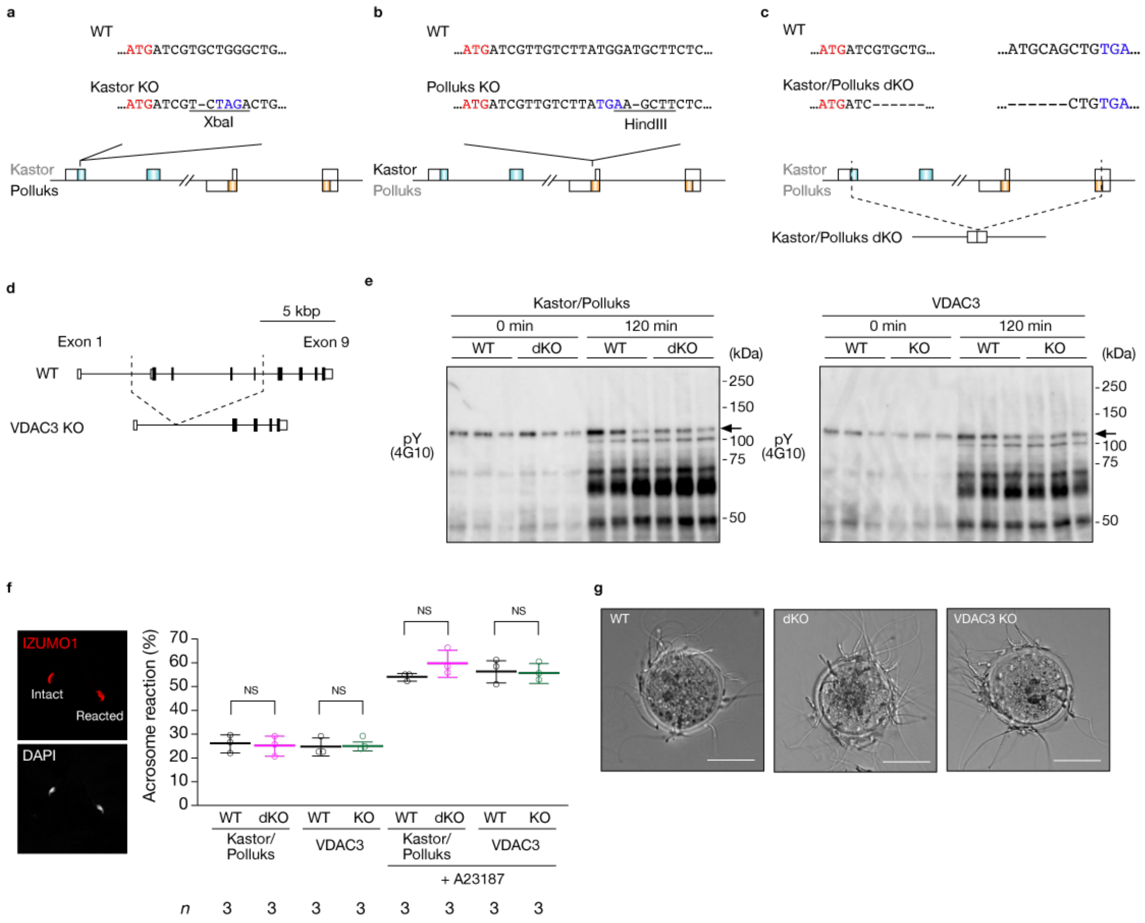

Supplementary Fig. 5 Generation and analysis of knockout (KO) mice. a–d Schematic representation of WT and mutant alleles for Kastor (**a**), Polluks (**b**), Kastor and Polluks (**c**), or VDAC3 (**d**) KO mice. Exons are denoted by boxes. ORFs of Kastor, Polluks, and VDAC3 are represented by blue, orange, and black boxes, respectively. For genotyping, 1-bp substitutions (G → A) that generated an XbaI site for Kastor KO and a HindIII site for Polluks KO were also introduced. Red and blue characters indicate initiation codons and stop codons, respectively. **e** Immunoblot analysis with antibodies to phosphotyrosine (pY, 4G10) for spermatozoa collected from the cauda epididymis of adult dKO or VDAC3 KO mice and incubated in mHTF medium for 0 or 2 h. Arrows indicate sperm hexokinase, which is constitutively tyrosine-phosphorylated and was used as an internal loading control. **f** Representative image of the acrosome reaction as revealed by IZUMO1 immunofluorescence (left), and the percentage of spermatozoa having undergone the acrosome reaction among total spermatozoa collected from mice of the indicated genotypes and incubated in capacitation medium with or without the Ca^{2+} ionophore A23187 (right). “Reacted” and “Intact” indicate

representative spermatozoa that did or did not undergo the acrosome reaction, respectively. Quantitative data are means ± s.d. for the indicated numbers of mice. NS (unpaired two-tailed Student's *t* test). **g** Bright-field images of the binding of spermatozoa isolated from mice of the indicated genotypes to the ZP of cumulus-free WT oocytes in vitro. Scale bars, 50 μm. Source data are provided as a Source Data file.

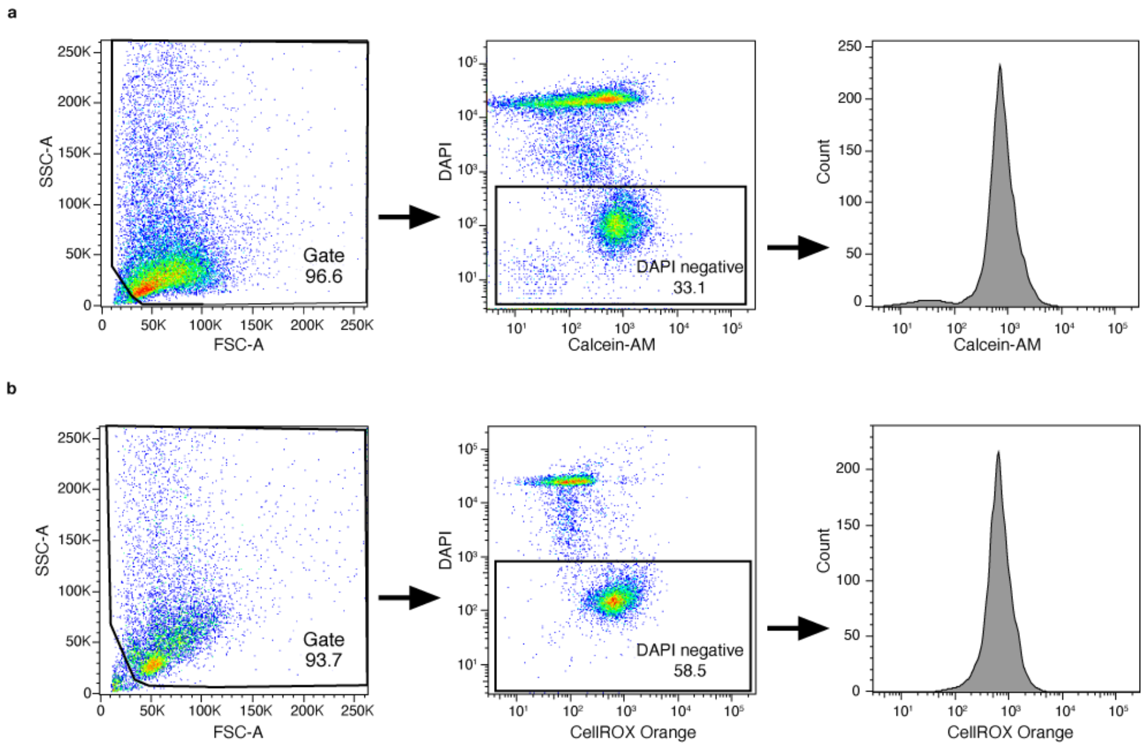

Supplementary Fig. 6 Gating strategy for flow cytometry. a Representative data for the mPTP assay (corresponding to Fig. 8h, i). **b** Representative data for the mitochondrial ROS assay (corresponding to Fig. 8k). FSC-A/SSC-A gates and DAPI-negative gates were used to exclude debris and dead cells, respectively.

UNCROPPED IMAGES OF WESTERN BLOT ANALYSIS

Supplementary Fig. 4e

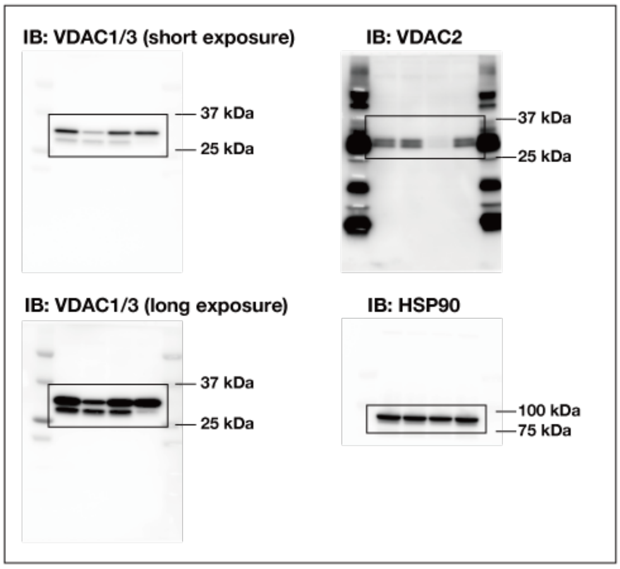

Supplementary Fig. 5e

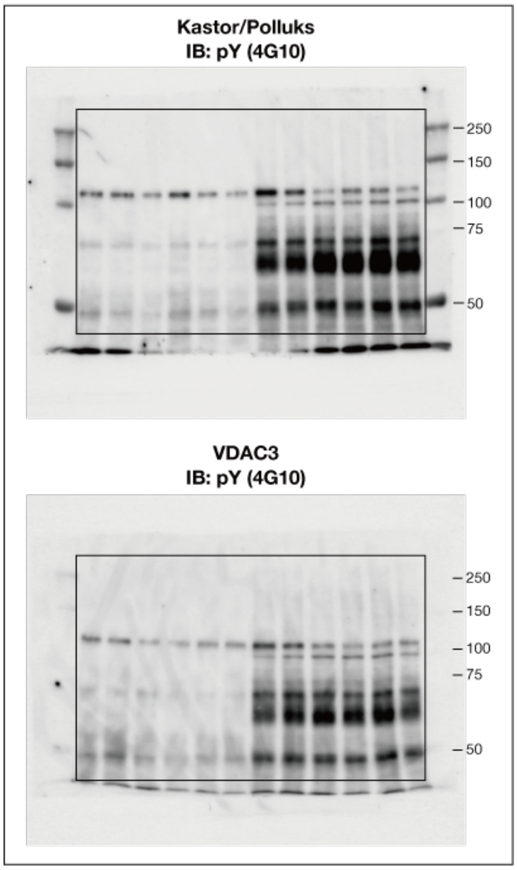

Supplement: Supplementary file 1 — Supplementary Information [file 41467_2022_28677_MOESM1_ESM.pdf]
