# Supplementary material for: Kastor and Polluks polypeptides encoded by a single gene locus cooperatively regulate VDAC and spermatogenesis
Source: Nat Commun. 2022 Feb 28;13:1071. doi: 10.1038/s41467-022-28677-y (PMC8885739; doi:10.1038/s41467-022-28677-y)
Supplement: Supplementary file 3 — Description of Additional Supplementary Files [file 41467_2022_28677_MOESM3_ESM.pdf]

**Description of Additional Supplementary Files**

**File name: Supplementary Data 1.**
Description: List of PhyloCSF scores and RNA expression levels for testis-specific lncRNAs.

**File name: Supplementary Data 2.**
Description: List of identified peptides derived from endogenous Kastor and Polluks in testis of *Kastor*$^{FLAG/+}$ or *Polluks*$^{FLAG/+}$ mice.

**File name: Supplementary Data 3.**
Description: iBAQ values for Kastor or Polluks binding proteins identified in testis of *Kastor*$^{FLAG/+}$ or *Polluks*$^{FLAG/+}$ mice.

**File name: Supplementary Data 4.**
Description: iBAQ values for Kastor or Polluks binding proteins identified in HEK293T cells transiently expressing Kastor-(C)FLAG or Polluks-(C)FLAG.

**File name: Supplementary Data 5.**
Description: Oligonucleotides and antibodies used in the present study.

**File name: Supplementary Movie 1.**
Description: Movement of spermatozoa collected from the cauda epididymis of WT mice as captured by a high-speed camera.

**File name: Supplementary Movie 2.**
Description: Movement of spermatozoa collected from the cauda epididymis of Kastor KO mice as captured by a high-speed camera.

**File name: Supplementary Movie 3.**
Description: Movement of spermatozoa collected from the cauda epididymis of Polluks KO mice as captured by a high-speed camera.

**File name: Supplementary Movie 4.**
Description: Movement of spermatozoa collected from the cauda epididymis of Kastor/Polluks dKO mice as captured by a high-speed camera.

**File name: Supplementary Movie 5.**

Description: Movement of spermatozoa collected from the cauda epididymis of VDAC3 KO mice as captured by a high-speed camera.
