# Supplementary material for: Kastor and Polluks polypeptides encoded by a single gene locus cooperatively regulate VDAC and spermatogenesis
Source: Nat Commun. 2022 Feb 28;13:1071. doi: 10.1038/s41467-022-28677-y (PMC8885739; doi:10.1038/s41467-022-28677-y)
Supplement: Supplementary file 14 — Reporting Summary [file 41467_2022_28677_MOESM14_ESM.pdf]

nature portfolio

Corresponding author(s): Keiichi I. Nakayama

Last updated by author(s): Jan 27, 2022

# Reporting Summary

Nature Portfolio wishes to improve the reproducibility of the work that we publish. This form provides structure for consistency and transparency in reporting. For further information on Nature Portfolio policies, see our Editorial Policies and the Editorial Policy Checklist.

## Statistics

For all statistical analyses, confirm that the following items are present in the figure legend, table legend, main text, or Methods section.

| n/a | Confirmed | |
|---|---|---|
| ☐ | ☒ | The exact sample size (*n*) for each experimental group/condition, given as a discrete number and unit of measurement |
| ☐ | ☒ | A statement on whether measurements were taken from distinct samples or whether the same sample was measured repeatedly |
| ☐ | ☒ | The statistical test(s) used AND whether they are one- or two-sided<br>*Only common tests should be described solely by name; describe more complex techniques in the Methods section.* |
| ☒ | ☐ | A description of all covariates tested |
| ☐ | ☒ | A description of any assumptions or corrections, such as tests of normality and adjustment for multiple comparisons |
| ☐ | ☒ | A full description of the statistical parameters including central tendency (e.g. means) or other basic estimates (e.g. regression coefficient) AND variation (e.g. standard deviation) or associated estimates of uncertainty (e.g. confidence intervals) |
| ☐ | ☒ | For null hypothesis testing, the test statistic (e.g. $F$, $t$, $r$) with confidence intervals, effect sizes, degrees of freedom and $P$ value noted<br>*Give P values as exact values whenever suitable.* |
| ☒ | ☐ | For Bayesian analysis, information on the choice of priors and Markov chain Monte Carlo settings |
| ☒ | ☐ | For hierarchical and complex designs, identification of the appropriate level for tests and full reporting of outcomes |
| ☒ | ☐ | Estimates of effect sizes (e.g. Cohen's *d*, Pearson's *r*), indicating how they were calculated |

*Our web collection on statistics for biologists contains articles on many of the points above.*

## Software and code

Policy information about availability of computer code

| | |
|---|---|
| Data collection | BD FACSVerse; StepOnePlus Real-Time system; XF24 extracellular flux analyzer; Orbitrap Velos Pro |
| Data analysis | ImageJ Fiji software (version 2.1.0); MaxQuant (version 1.6.3.4); JMP (version 16); Mascot; FlowJo V10; Microsoft Excel 365 (version 16.57); TMpred (https://embnet.vital-it.ch/software/TMPRED_form.html); ProtScale (https://web.expasy.org/protscale/); tBlastN (https://blast.ncbi.nlm.nih.gov/Blast.cgi) |

For manuscripts utilizing custom algorithms or software that are central to the research but not yet described in published literature, software must be made available to editors and reviewers. We strongly encourage code deposition in a community repository (e.g. GitHub). See the Nature Portfolio guidelines for submitting code & software for further information.

## Data

Policy information about availability of data

All manuscripts must include a data availability statement. This statement should provide the following information, where applicable:

- Accession codes, unique identifiers, or web links for publicly available datasets
- A description of any restrictions on data availability
- For clinical datasets or third party data, please ensure that the statement adheres to our policy

The MS data have been deposited with the ProteomeXchange Consortium (http://proteomecentral.proteomexchange.org) via the JPOST partner repository under the data set identifier PXD027126 [http://proteomecentral.proteomexchange.org/cgi/GetDataset?ID=PXD027126]. Data on lncRNA expression levels in testis were obtained from the NCBI database (https://www.ncbi.nlm.nih.gov).

# Field-specific reporting

Please select the one below that is the best fit for your research. If you are not sure, read the appropriate sections before making your selection.

☒ Life sciences ☐ Behavioural & social sciences ☐ Ecological, evolutionary & environmental sciences

For a reference copy of the document with all sections, see nature.com/documents/nr-reporting-summary-flat.pdf

# Life sciences study design

All studies must disclose on these points even when the disclosure is negative.

| | |
|---|---|
| Sample size | No statistical methods were used to predetermine sample size in this study, but the sample-size were determined based on previous report (Miyata et al. Science 350.6259 (2015): 442-445.). |
| Data exclusions | No data was excluded. |
| Replication | All experiments were conducted with multiple biological replicates shown in figures, figure legends or methods. For almost all experiments, more than 3 biologically independent replicates were analyzed. |
| Randomization | All individual mice or samples were randomly allocated to the experimental groups. |
| Blinding | The study was essentially not blinded because it was necessary for investigators to be aware of the information of mouse genotypes, conditions and cell types to be compared. |

# Reporting for specific materials, systems and methods

We require information from authors about some types of materials, experimental systems and methods used in many studies. Here, indicate whether each material, system or method listed is relevant to your study. If you are not sure if a list item applies to your research, read the appropriate section before selecting a response.

## Materials & experimental systems

| n/a | Involved in the study | |
|---|---|---|
| ☐ | ☒ | Antibodies |
| ☐ | ☒ | Eukaryotic cell lines |
| ☒ | ☐ | Palaeontology and archaeology |
| ☐ | ☒ | Animals and other organisms |
| ☒ | ☐ | Human research participants |
| ☒ | ☐ | Clinical data |
| ☒ | ☐ | Dual use research of concern |

## Methods

| n/a | Involved in the study | |
|---|---|---|
| ☒ | ☐ | ChIP-seq |
| ☐ | ☒ | Flow cytometry |
| ☒ | ☐ | MRI-based neuroimaging |

## Antibodies

Antibodies used

Rat monoclonal antibodies to Kastor and to Polluks were generated in this study as described in methods section.

Antibody name/Supplier, Cat#/Dilution

[Immunoblot]
Mouse monoclonal anti-α-tubulin/Thermo Fisher Scientific, 13-8000/1:250
Mouse monoclonal anti-COXIV/Thermo Fisher Scientific, A21348/1:1000
Mouse monoclonal anti-FLAG/Sigma, F1804/1:1000
Rabbit polyclonal anti-DDDDK/Medical & Biological Laboratories, PM020/1:1000
Rabbit polyclonal anti-VDAC2/ProteinTech, 11663-1-AP/1:1000
Rabbit polyclonal anti-COXIV/ProteinTech, 11242-1-AP/1:1000
Mouse monoclonal anti-VDAC/Abcam, ab14734/1:1000
Rabbit monoclonal anti-VDAC1/Abcam, ab154856/1:1000
Rabbit monoclonal anti-TIM44/Abcam, ab194829/1:1000
Rabbit monoclonal anti-TOM20/Abcam, ab186735/1:1000
Mouse monoclonal anti-TOM20/Santa Cruz Biotechnology, sc-17764/1:250
Mouse monoclonal anti-GAPDH/Enzo Life Sciences, ADI-CSA-335-E/1:1000
Mouse monoclonal anti-HSP90/BD Biosciences, 610419/1:1000
Mouse monoclonal anti-phosphotyrosine/Sigma, 05321/1:10000
Anti-Mouse IgG, HRP–conjugated/Promega, W4021/1:20000

Anti-Rabbit IgG, HRP–conjugated/Promega, W4011/1:20000
Goat anti-rat IgG (H+L) secondary antibody/Thermo Fisher Scientific, 31470/1:20000

[Immunofluorescence]
Mouse monoclonal anti-FLAG/Sigma, F1804/1:500
Rabbit polyclonal anti-FLAG/Sigma, F7425/1:500
Rabbit polyclonal anti-DDDDK/Medical & Biological Laboratories, PM020/1:200
Rabbit monoclonal anti-TOM20/Abcam, ab186735/1:50
Rat monoclonal anti-IZUMO1/BioAcademia, 73-045/1:500
Mouse monoclonal anti-SYCP3/Santa CruZ Biotechnology, sc-74569/1:50
Mouse monoclonal anti-Vimentin/Santa CruZ Biotechnology, sc-373717/1:50
Mouse monoclonal anti-3β-HSD/Santa CruZ Biotechnology, sc-515120/1:50
Mouse monoclonal anti-E-cadherin/Santa CruZ Biotechnology, sc-8426/1:50
Alexa Fluor 488–conjugated goat anti-rabbit IgG/Thermo Fisher Scientific, A-11034/1:500
Alexa Fluor 546–conjugated goat anti-rabbit IgG/Thermo Fisher Scientific, A-11035/1:500
Alexa Fluor 488–conjugated goat anti-mouse IgG/Thermo Fisher Scientific, A-11029/1:500
Alexa Fluor 546–conjugated goat anti-mouse IgG/Thermo Fisher Scientific, A-11030/1:500
Alexa Fluor 546–conjugated goat anti-rat IgG/Thermo Fisher Scientific, A-11081/1:500

[in situ hybridization]
Anti-Digoxigenin-AP, Fab fragments/Roche, 11093274910

Validation

Rat monoclonal anti-kastor and anti-polluks antibodies generated in this study were confirmed to specifically recognize Kastor and Polluks, respectively, using testis and sperm from Kastor KO, Polluks KO, and Kasotr/Polluks dKO mice as shown in fig. 5A.

For all antibodies used in this study, the websites of corresponding suppliers are listed below. Please see them to check general validation, relevant citations or application of each antibody.

Mouse monoclonal anti-α-tubulin/Thermo Fisher Scientific, 13-8000 (https://www.thermofisher.com/antibody/product/alpha-Tubulin-Antibody-clone-TU-01-Monoclonal/13-8000)
Mouse monoclonal anti-COXIV/Thermo Fisher Scientific, A21348 (https://www.thermofisher.com/antibody/product/OxPhos-Complex-IV-subunit-IV-Antibody-clone-20E8C12-Monoclonal/A21348)
Mouse monoclonal anti-FLAG/Sigma, F1804 (https://www.sigmaaldrich.com/JP/en/product/sigma/f1804)
Rabbit polyclonal anti-DDDDK/Medical & Biological Laboratories, PM020 (https://ruo.mbl.co.jp/bio/dtl/A/?pcd=PM020)
Rabbit polyclonal anti-VDAC2/ProteinTech, 11663-1-AP (https://www.ptglab.co.jp/products/VDAC2-Antibody-11663-1-AP.htm)
Rabbit polyclonal anti-COXIV/ProteinTech, 11242-1-AP (https://www.ptglab.co.jp/products/COX4I1-Antibody-11242-1-AP.htm)
Mouse monoclonal anti-VDAC/Abcam, ab14734 (https://www.abcam.co.jp/vdac1porin--vdac3-antibody-20b12af2-ab14734.html)
Rabbit monoclonal anti-VDAC1/Abcam, ab154856 (https://www.abcam.co.jp/vdac1porin--vdac2-antibody-epr10852b-mitochondrial-loading-control-ab154856.html)
Rabbit monoclonal anti-TIM44/Abcam, ab194829 (https://www.abcam.co.jp/tim44-antibody-epr16821-ab194829.html)
Rabbit monoclonal anti-TOM20/Abcam, ab186735 (https://www.abcam.co.jp/tomm20-antibody-epr15581-54-mitochondrial-marker-ab186735.html)
Mouse monoclonal anti-TOM20/Santa Cruz Biotechnology, sc-17764 (https://www.scbt.com/ja/p/tom20-antibody-f-10)
Mouse monoclonal anti-GAPDH/Enzo Life Sciences, ADI-CSA-335-E (https://www.enzolifesciences.com/ADI-CSA-335/gapdh-monoclonal-antibody-1d4/)
Mouse monoclonal anti-HSP90/BD Biosciences, 610419 (https://www.bdbiosciences.com/ja-jp/products/reagents/microscopy-imaging-reagents/immunofluorescence-reagents/purified-mouse-anti-hsp90.610419)
Mouse monoclonal anti-phosphotyrosine/Sigma, 05321 (https://www.sigmaaldrich.com/JP/en/product/mm/05321)
Anti-Mouse IgG, HRP–conjugated/Promega, W4021 (https://www.promega.jp/products/protein-detection/primary-and-secondary-antibodies/anti_mouse-igg-h-and-l-hrp-conjugate/?catNum=W4021)
Anti-Rabbit IgG, HRP–conjugated/Promega, W4011 (https://www.promega.jp/products/protein-detection/primary-and-secondary-antibodies/anti-rabbit-igg-h-and-l-hrp-conjugate/?catNum=W4011)
Goat anti-rat IgG (H+L) secondary antibody/Thermo Fisher Scientific, 31470 (https://www.thermofisher.com/antibody/product/Goat-anti-Rat-IgG-H-L-Secondary-Antibody-Polyclonal/31470)
Rabbit polyclonal anti-FLAG/Sigma, F7425 (https://www.sigmaaldrich.com/JP/en/product/sigma/f7425?gclid=EAIaIQobChMIv8Cq7djO9QIVETdgCh1xKA7DEAAYASAAEgLnLfD_BwE)
Rat monoclonal anti-IZUMO1/BioAcademia, 73-045 (https://www.bioacademia.co.jp/html/upload/save_image/E73-045%20anti-IZUMO1%20antibody,%20rat%20monoclonal%20(.pdf)
Mouse monoclonal anti-SYCP3/Santa CruZ Biotechnology, sc-74569 (https://www.scbt.com/ja/p/scp-3-antibody-d-1)
Mouse monoclonal anti-Vimentin/Santa CruZ Biotechnology, sc-373717 (https://www.scbt.com/ja/p/vimentin-antibody-e-5)
Mouse monoclonal anti-3β-HSD/Santa CruZ Biotechnology, sc-515120 (https://www.scbt.com/ja/p/3beta-hsd-antibody-a-1)
Mouse monoclonal anti-E-cadherin/Santa CruZ Biotechnology, sc-8426 (https://www.scbt.com/ja/p/e-cadherin-antibody-g-10)
Alexa Fluor 488–conjugated goat anti-rabbit IgG/Thermo Fisher Scientific, A-11034 (https://www.googleadservices.com/pagead/aclk?sa=L&ai=DChcSEwiJ1cm72c71AhWPXWAKHYDiA8wYABAAGgJ0bQ&ae=2&ohost=www.google.com&cid=CAASE-RoRmuvjVysQEVGCy4LtBkIRrY&sig=AOD64_0_F22w3wIogSTrQ0v3SJ4FTI3EiQ&q&adurl&ved=2ahUKEwi8wMC72c71AhXNslYBHbu-C-gQ0Qx6BAgCEAE&dct=1)
Alexa Fluor 546–conjugated goat anti-rabbit IgG/Thermo Fisher Scientific, A-11035 (https://www.thermofisher.com/antibody/product/Goat-anti-Rabbit-IgG-H-L-Highly-Cross-Adsorbed-Secondary-Antibody-Polyclonal/A-11035)
Alexa Fluor 488–conjugated goat anti-mouse IgG/Thermo Fisher Scientific, A-11029 (https://www.thermofisher.com/antibody/product/Goat-anti-Mouse-IgG-H-L-Highly-Cross-Adsorbed-Secondary-Antibody-Polyclonal/A-11029)

Alexa Fluor 546–conjugated goat anti-mouse IgG/Thermo Fisher Scientific, A-11030 (https://www.thermofisher.com/antibody/product/Goat-anti-Mouse-IgG-H-L-Highly-Cross-Adsorbed-Secondary-Antibody-Polyclonal/A-11030)
Alexa Fluor 546–conjugated goat anti-rat IgG/Thermo Fisher Scientific, A-11081 (https://www.thermofisher.com/antibody/product/A-11081.html?ef_id=EAIaIQobChMIiNeF4NnO9QIVuNxMAh3UMwZWEAAYASAAEgIaifD_BwE:G:s&s_kwcid=AL!3652!3!516608152200!!!g!!&cid=bid_pca_aus_r01_co_cp1359_pjt0000_bid00000_0se_gaw_dy_pur_con&gclid=EAIaIQobChMIiNeF4NnO9QIVuNxMAh3UMwZWEAAYASAAEgIaifD_BwE)
Anti-Digoxigenin-AP, Fab fragments/Roche, 11093274910 (https://www.sigmaaldrich.com/JP/en/product/roche/11093274910)

# Eukaryotic cell lines

Policy information about cell lines

| | |
|---|---|
| Cell line source(s) | HEK293T, HeLa, Neuro2A, and SP2 cells were obtained from ATCC. |
| Authentication | All cell lines were purchased directly from ATCC. No additional authentication was performed by the authors. |
| Mycoplasma contamination | Cells were tested for mycoplasma using the MC-210 (KAC, Cat #88101-2) and PCR analysis. All cells were confirmed to be free of mycoplasma. |
| Commonly misidentified lines (See ICLAC register) | Commonly misidentified cell line was not used. |

# Animals and other organisms

Policy information about studies involving animals; ARRIVE guidelines recommended for reporting animal research

| | |
|---|---|
| Laboratory animals | Female C57BL/6 mice (3-8 weeks of age) and female B6D2F1/Jcl (C57BL/6N Jcl × DBA/2N Jcl, 8 weeks of age) mice were purchased from CLEA Japan. Kastor FLAG-tag knock-in mice, Polluks FLAG-tag knock-in mice, Kastor KO mice, Polluks KO mice, Kastor/Polluks dKO mice, and VDAC3 KO mice were generated as described in methods section and 8-56 weeks old male mice were used in our experiments. All mice were housed in the specific pathogen–free animal facility at Kyushu University in accordance with institutional guidelines under the following conditions: 22°C ambient temperature, 50–60% humidity, 12 h dark/light cycle, and free access to water and rodent chow CA-1 (CLEA Japan). |
| Wild animals | No wild animals were used. |
| Field-collected samples | No field collected samples were used in the study. |
| Ethics oversight | All animal experiments were approved by the animal ethics committee of Kyushu University (A20-169-0) and were conducted in compliance with the university guidelines and regulations for animal experimentation. |

Note that full information on the approval of the study protocol must also be provided in the manuscript.

# Flow Cytometry

## Plots

Confirm that:

- [x] The axis labels state the marker and fluorochrome used (e.g. CD4-FITC).
- [x] The axis scales are clearly visible. Include numbers along axes only for bottom left plot of group (a 'group' is an analysis of identical markers).
- [x] All plots are contour plots with outliers or pseudocolor plots.
- [x] A numerical value for number of cells or percentage (with statistics) is provided.

## Methodology

| | |
|---|---|
| Sample preparation | Spermatozoa were collected from cauda epididymis and cultured in mHTF medium at 37°C. For mPTP assay, spermatozoa were incubated with 0.03 µM calcein-AM and 4.8 µM cobalt chloride for 15 min, and immediately analyzed. For ROS analysis, spermatozoa were incubated with 1 µM CellROX Orange (Invitrogen) for 30 min, and immediately analyzed. |
| Instrument | BD FACSVerse Flow Cytometer (Becton, Dickinson and Company) |
| Software | FlowJo V10; Microsoft Excel |
| Cell population abundance | 10,000 or 20,000 sperm cell events were collected for each samples. |
| Gating strategy | Debris were discriminated using FSC/SSC plot followed by exclusion of dead cells using DAPI. MFI of calcein-AM or CellROX Orange were calculated using the resultant cells. |

- [x] Tick this box to confirm that a figure exemplifying the gating strategy is provided in the Supplementary Information.
